# Diversity Oriented Design of Various Benzophenone Derivatives and Their *in Vitro* Antifungal and Antibacterial Activities

**DOI:** 10.3390/molecules16119739

**Published:** 2011-11-23

**Authors:** Li Sun, Jie Wu, Min Luo, Xiaoli Wang, Man Pan, Zhaopin Gou, Dequn Sun

**Affiliations:** Marine College, Shandong University, Weihai Wenhua West Road, No.180, Weihai 264209, China

**Keywords:** benzophenone derivatives, bioactivity, phytopathogenic fungi, vibrio

## Abstract

A series of new substituted benzophenone derivatives was designed, synthesized and screened for their antifungal and antibacterial activities. The bioassays indicated that most of the synthesized compounds showed some antifungal activity against the tested phytopathogenic fungi, but lower antibacterial activities towards the five vibrios isolated from marine sources. The preliminary structure activity relationship (SAR) of the compounds was also discussed.

## 1. Introduction

**Figure 1:**
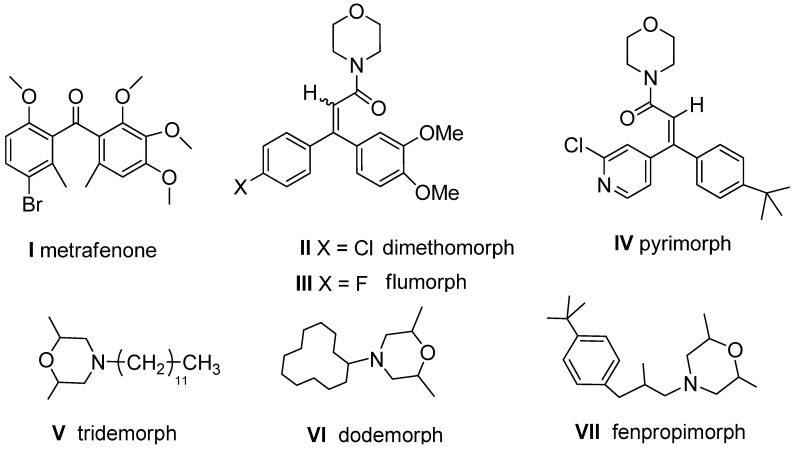
The chemical structures of several commercial fungicides.

Resistance and cross-resistance are easily found in the field when a class of agrochemicals has been used for a long time [1], thus exploring new agrochemical candidates with different modes of action is always meaningful. Metrafenone (**I**, Figure 1) is a novel benzophenone-derived fungicide [2] recently registered in several countries for control of powdery mildews in different crops [3,4] and eye spot in cereals [5]. It possess a different mode of action that might interfere with processes which are essential to establish and maintain polar action organization [6]. The carboxylic acid amide (CAA) fungicides, dimethomorph (**II**, Figure 1) and flumorph (**III**, Figure 1) [7,8] are derivatives of benzophenone and are common agrochemicals in the field in China nowadays. They have been widely used for years to control the diseases caused by Oomycete foliar plant pathogens (e.g., *Phytophthora infestans* (Mont.) de Bary and *Peronosporaceae*) with the mode of inhibiting cellulose synthesis [8]. Recently, Qin [9] synthesized a novel fungicide, pyrimorph (**IV**, Figure 1), which was actually an analog of **II** and **III** and exhibited excellent activity against oomycetes. Dimethomorph, flumorph and pyrimorph have both a benzophenone skeleton and a morpholine ring in their chemical structures. The morpholine group is likely to be a pharmacophore in fungicidal chemicals since it exists in a number of other commercial fungicides, such as tridemorph (**V**, Figure 1), dodemorph (**VI**, Figure 1) and fenpropimorph (**VII**, Figure 1) [10].

**Scheme 1:**
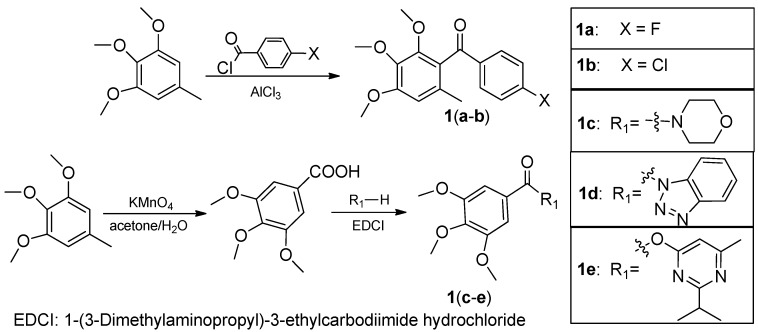
The synthetic routes of compounds **1a**–**e**.

**Scheme 2:**
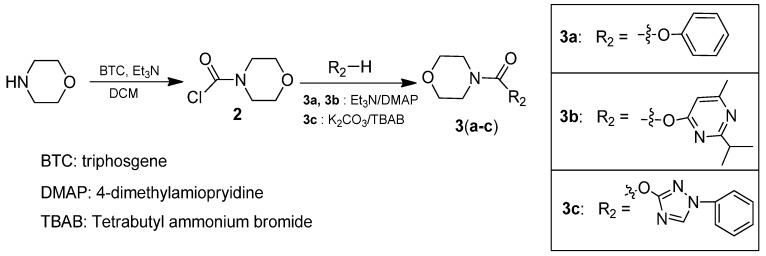
The synthetic routes of compounds **3a**–**c**.

**Scheme 3:**
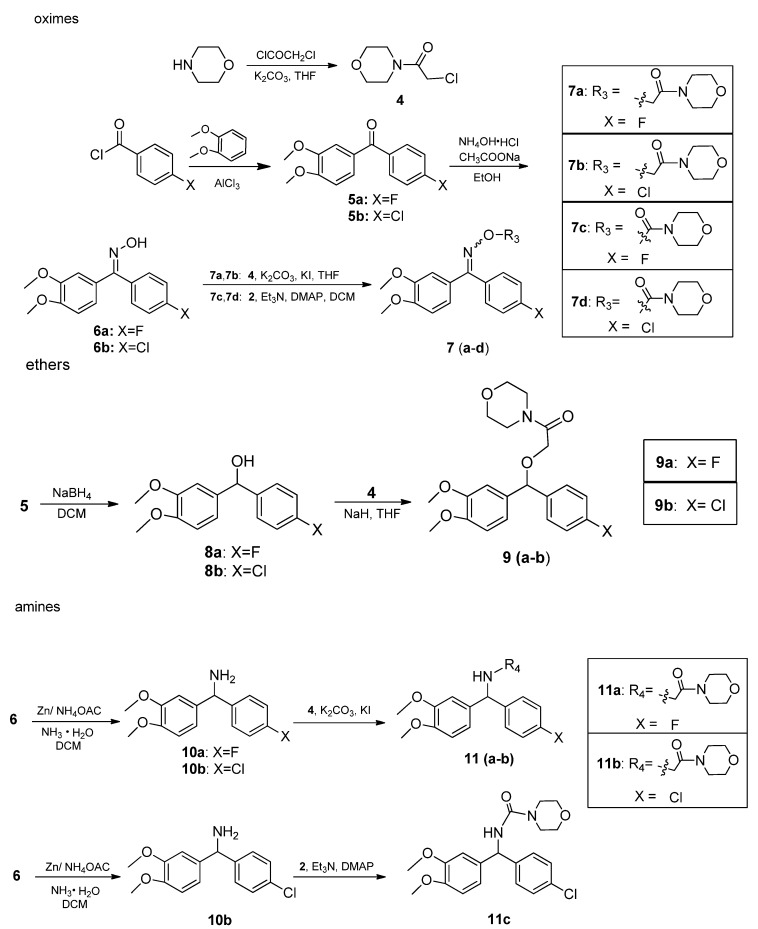
The synthetic routes of compounds **7**, **9** and **11**.

To the best of our knowledge, studies concerning metrafenone analogs as antifungal or antibacterial agents have seldom been reported. In an attempt to screen for new agrochemicals and predict their relatively detailed SAR, a diverse series of benzophenone derivatives, some of which contained a morpholine group, were designed on the basis of bioisosterism principles [11] and synthesized (Scheme 1, Scheme 2 and Scheme 3). Among the compounds, twelve new compounds and five known compounds were tested on the phytopathogenic fungi and vibrios. Compounds **1b** [12] and **3a** [13] were synthesized before, but no studies on their bioactivity were reported. The silica gel-mediated synthesis and cytotoxic activity of **1c** were reported [14]. Compound **1d** was studied by Jie [15] with respect to its potential antiproliferative activity. Compounds **1e** and **3b** were already reported in our previous work [16].

As suggested by the SAR of metrofenone, one benzene ring of metrafenone was replaced by other potentially active moieties, such as aryl, aryloxy or aromatic heterocycles to give the first kind of new benzophenone derivatives **1a**–**e** (Scheme 1). The second series of novel substituted benzophenone derivatives **3a**, **3b** and **3c**, containing morpholine groups, were synthesized as shown in Scheme 2.

To learn more about SAR of the compounds containing benzophenone skeletons and morpholine groups, the new oxime derivatives **7** were designed by replacing the C atom of dimethomorph or flumorph with an isosteric N atom, and after reduction of the double bond, ethers **9** and amines **11** were obtained (Scheme 3).

## 2. Results and Discussion

### 2.1. Chemistry

As shown in Scheme 1, **1a** and **1b** were synthesized via the classic Friedel-Crafts reaction from the appropriate benzoyl chlorides and 3,4,5-trimethoxytoluene. Compound **1b** was also synthesized using a different method from the literature [12] in similar yield. Oxidation of the methyl group on 3,4,5-trimethoxytoluene followed by condensation with morpholine, 6-hydroxy-2-isopropyl-4-methylpyrimidine and 1*H*-benzotriazole gave **1c**, **1d** and **1e**, respectively. Compounds **3a** and **3c** were prepared using Et_3_N and DMAP in DCM (73% and 72% respectively) and **3c** was obtained using K_2_CO_3_ and TBAB in DCM in a yield of 63%.

The synthetic routes to **7**, **9** and **11** are outlined in Scheme 3. The benzophenone skeleton (compound **5**) was synthesized from the 4-halosubstituted benzoyl chloride and 1,2-dimethoxybenzene starting materials via the classic Friedel-Crafts reaction. Compound **6** was obtained (in 96.7% yield) through coupling of **5** with NH_4_OH·HCl using CH_3_COONa as base and the generated CH_3_COOH as catalyst. The etherification reaction between compound **6** and **4** gave **7a** and **7b**, the esterification reaction between **6** and **2** gave **7c** and **7d** under the corresponding conditions with good conversion.

The substituted benzophenone **5** was reduced to diphenyl methanol **8** with NaBH_4_ in DCM cleanly and almost quantitatively (98%). Etherification of **8** with 4-(2-chloroacetyl) morpholine (**4**) gave **9a** and **9b** in yields of 91% and 84% respectively using NaH in THF. Reductive ammoniation of compound **6** with zinc dust in 30% ammonia afforded amine **10**, which was treated with **4** in the presence of K_2_CO_3_ and KI to give **11a** and **11b** or with **2** using Et_3_N and DMAP to give **11c** with yields higher than 70%.

### 2.2. Bioactivity

All of the tested compounds had the purity of more than 95%. The oximes were tested using a mixture of *(Z)*- and *(E)*-isomers and the enantiomers were tested as racemic mixtures. The bioactivities towards phytopathogenic fungi and five vibrios were evaluated. The phytopathogenic fungi chosen included *Alternaria kukuchiana* (AK), *Alternaria mali* (AM), *Botrytis cinerea* (BC), *Bipolaris maydi* (BM), *Cercospora arachidicola* (CA), *Gibberella zeae* (GZ), *Gibberella fujikuroi* (GF), *Macrophoma kuwatsukai* (MK), *Rhizoctonia solani* (RS), *Sclerotinia sclerotiorum* (SS), *Thanatephorus cucumeris* (Frank), Domk (TC) and *Fusarium oxysporum f. sp. Vasinfectum*. All these fungi are different typical genera, often found in the Chinese agro-ecosystem. The five tested vibrios (*Vibrio Parahaemolyticus*, *Vibrio harvyi*, *Vibrio anguillarum*, *Vibrio alginolyticus*, *Vibrio vulnificus*) were isolated from marine organism. The bioactivity results are outlined in Tables respectively.

#### 2.2.1. Antifungal Activity to Phytopathogenic Fungi

##### 2.2.1.1. Antifungal Activity of Compounds **1** to ten Phytopathogenic Fungi

**Table 1:** The antifungal activity of the synthesized compounds **1**.

Compd. No.	Fungicidal activities (50 μg/mL, inhibition rate %)									
	AK	BC	BM	CA	GF	GZ	MK	RS	SS	TC
**1a**	45.5	7.4	34.8	6.7	33.3	11.1	0	66.7	0	21.6
**1b**	53.6	11.1	35.1	4.5	39.2	7.8	0	68.3	0	33.3
**1c**	22.7	11.1	8.7	20	6.7	3.7	37.5	44.4	13.3	7.8
**1d **	22.7	7.4	13.0	0	20	3.7	37.5	61.9	6.7	3.9
**1e**	31.8	14.8	26.1	6.7	20	7.4	40.6	63.5	66.7	9.8
**Flu.**	18.2	7.4	17.4	0	13.3	7.4	21.9	61.9	0	9.8
**Dim.**	22.7	18.5	17.4	0	20	0	6.3	68.3	20	23.5

**Flu. =** flumorph, **Dim. =** dimethomorph.

Among compounds **1** (Table 1), more than one compound showed similar or better activity than flumorph and dimethomorph towards the tested fungi. Compounds **1a** and **1b** were more potent to most of the tested fungi belonging to the different genera. Both **1c** and **1e** had certain inhibitory activity against all of the tested fungi. Compound **1e** containing a 3,4,5-trimethoxybenzene ring and pyrimidine group exhibited good activity compared to controls, especially to MK and SS, while **1e** showed remarkable inhibition rates that were about 1-fold and 2-fold higher than controls, respectively (40.6%/21.9%, 66.7%/20%). It’s interesting that compounds **1c**-**1e** all had activity on MK and SS, while **1a** and **1b** had no effect, which suggested that N-containing heterocycles might be the necessary groups to inhibit MK and SS. Compounds **1a**, **1b** and **1e** could be better fungicidal candidates since they showed better inhibition rates and relatively good broad-spectrum activity towards the tested fungi of diverse genera.

##### 2.2.1.2. Antifungal Activity of Compounds **3** to Nine Phytopathogenic Fungi

After the 3,4,5-trimethoxybenzene rings in **1c**, **1d** and **1e** were replaced by morpholine groups to obtain new compounds, the structures of which are very different from metrofenone and flumorph or dimethomorph, compounds **3a**–**c** showed similar or higher activity to nine fungi compared with flumorph and dimethomorph. Compounds **3b** and **3c** showed better broad-spectrum activity. According to the SAR discussion on compounds **1** and **3**, a trimethoxybenzene ring was favorable for increased fungicidal activity.

**Table 2:** The antifungal activities of compounds **3**.

Compd. No.	Fungicidal activities (50 μg/mL, inhibition rate %)								
	AK	BC	BM	CA	GF	GZ	MK	RS	TC
**3a**	22.7	7.4	17.4	6.7	20	22.2	21.9	65.1	0
**3b **	40.9	18.5	17.4	6.7	6.7	7.4	31.3	61.9	19.6
**3c**	13.6	14.8	30.4	6.7	13.3	7.4	28.1	57.1	23.5
**Flu.**	18.2	7.4	17.4	0	13.3	7.4	21.9	61.9	9.8
**Dim.**	22.7	18.5	17.4	0	20	0	6.3	68.3	23.5

##### 2.2.1.3. Antifungal Activity of Compounds **7**, **9** and **11** to ten Phytopathogenic Fungi

As shown in Table 3, compounds **7c** and **11a** displayed better wide-spectrum activity than the controls. Compound **9b** showed significant inhibition of endophytic *Alternaria* spp. fungi such as AK and AM (27.3% and 57.1%, respectively).

**Table 3:** The antifungal activities of **7**, **9** and **11**.

Compd. No.	Fungicidal activities (50 μg/mL, inhibition rate %)									
	AK	AM	BC	BM	CA	GF	GZ	MK	RS	TC
**7a**	18.2	0	7.4	13.0	20	20	7.4	12.5	68.3	0
**7b**	22.7	0	14.8	13.0	6.7	20	11.1	25.0	61.9	3.9
**7c**	22.7	7.1	11.1	26.1	20	6.7	7.4	18.8	65.1	5.9
**7d **	27.3	0	3.7	34.8	6.7	20	14.8	0	61.9	27.5
**9a**	18.2	14.3	3.7	17.4	6.7	20	7.4	18.8	63.5	0
**9b**	27.3	57.1	3.7	13.0	0	6.7	0	18.8	63.5	2.0
**11a**	13.6	7.1	22.2	13.0	6.7	13.3	14.8	6.3	54.0	2.0
**11b**	18.2	0	7.4	13.0	13.3	13.3	0	15.6	54.0	0
**11c**	18.2	14.3	11.1	13.0	0	13.3	3.7	40.6	58.7	15.7
**Flu.**	18.2	21.4	7.4	17.4	0	13.3	7.4	21.9	61.9	9.8
**Dim.**	22.7	7.1	18.5	17.4	0	20	0	6.3	68.3	23.5

Replacement of the F atom on the benzene ring of **7a**, **7c**, **9a**, **11a** with Cl (**7b**, **7d**, **9b**, 1**1b**) led to a lightly increase in the inhibition of AK (18.2%/27.3%, 13.6%/18.2%, 18.2%, 22.7%, 22.7%/27.3%, respectively). Introduction of a -CH_2_ into the bridge between the morpholine group and the substituted benzophenone skeleton was favorable to the antifungal activity against AM; for example, **9b** was much more potent (57.1%) than dimethomorph (7.1%) and flumorph (21.4%); with respect to CA, most of the compounds showed activities, except **9b**, **11c** (0%), while flumorph and dimethomorph had no effect. Among the tested compounds, derivatives containing C=N, F and morpholine moieties such as **7a**, **7c** exhibited relatively higher inhibition rates (20%, 20%) than others (13.3%, 6.7% or 0) towards CA.

##### 2.2.1.4. Antifungal Activity Towards *Fusarium oxysporum f. sp. Vasinfectum*

*Fusarium wilt*, the “cancer” of cotton, caused by *Fusarium oxysporum f. sp. vasinfectum* is a global and serious threat to cotton, which is a high-value crop [17]. The pesticides carbendazim and dimethomorph are the two main chemicals used widely to prevent *Fusarium wilt* of cotton in the field in China, therefore carbendazim and dimethomorph were used as positive controls, and the antifungal activity of compounds **7b**–**d** to *Fusarium oxysporum f. sp. vasinfectum* was screened by the similar poisoned food technique at the concentrations of 500, 250 and 125 μg/mL, respectively (Table 4). Compounds **7b**–**d** all have higher activity than dimethomorph at different concentrations and this result revealed that replacing the C atom in dimethomorph with an isosteric atom N results in higher activity to *Fusarium wilt*; furthermore, inserting one CH_2_ the between carbonyl and oxygen in **7b** enhanced the activity, especially at the higher concentration. The Cl substituted compound **7d** is more potent than the F substituted compound **7c**.

It was interesting that **7b** showed better inhibition at the concentration of 500 μg/mL but lower activity than carbendazim at the concentrations of 250 and 125 μg/mL, indicating that **7b** might have a lower concentration than carbendazim when the inhibition rate reached to 100%. It might be better choice than carbendazim and represent another type of compound to prevent *Fusarium wilt* since its structure is totally different from carbendazim and might possess a different mode of inhibitory action.

**Table 4:** The inhibition rates to *Fusarium oxisporum f. sp. Vasinfectum.*

500 μg/mL		250 μg/mL		125 μg/mL	
Compd.	Inhibition rate (%)	Compd.	Inhibition rate (%)	Compd.	Inhibition rate (%)
**Carb.**	44.2	**Carb.**	39.3	**Carb.**	33.0
**Dim.**	12.5	**Dim.**	5.9	**Dim.**	3.1
**7b**	50.0	**7b**	19.7	**7b**	5.8
**7c**	27.4	**7c**	10.6	**7c**	3.9
**7d**	33.1	**7d**	13.3	**7d**	4.7

Carb.: carbendazim.

#### 2.2.2. Antibacterial Activity Towards Five Vibrios

Vibrios cause vibriosis, a common disease in marine aquaculture worldwide and a number of species are also human pathogens [18]. Antibiotics are widely used to treat vibrios and the most potent compounds are used to control vibriosis, which increases the resistance of the bacteria to commercially available antibiotics [19,20], in addition to their negative impact on the environment and residues in cultured marine animals. Nowadays, though many studies focus on the bacterial sensitivity to the extracts from seaweed or herbal plants, and discovery of the bacterial biocontrol agents [21], there are few reports about screening of synthetic compounds as potential bactericides which have good activity against vibrios. Therefore, the synthesized compounds were tested on five vibrios by simplified disc diffusion method [22]. Unfortunately, they exhibited different levels of inhibitory effects against the five vibrios at a relatively high concentration and were all much less effective than streptomycin and gentamicin sulfate.

## 3. Experimental

### 3.1. Chemistry

#### 3.1.1. General

Anhydrous solvents were distilled prior to use. All reactions using air- or moisture-sensitive reagents were conducted under an inert nitrogen atmosphere. The products were purified by column chromatography using silica gel (200–300 mesh). Melting points of the products were determined in open capillary tubes and are uncorrected. The IR spectra were recorded on a Bruker VERTEX 70 FT-IR instrument. ^1^H-NMR spectra were recorded on Varian-400 at r.t. using TMS as an internal reference. Mass spectra were recorded with a JEOL MS-D 300 mass spectrometer. Elemental analysis was performed on a Carlo-1106 model automatic instrument.

#### 3.1.2. Syntheses

*(4-Fluorophenyl)(2,3,4-trimethoxy-6-methylphenyl)methanone* (**1a**). A suspension of AlCl_3_ (0.52 g, 3.9 mmol) in DCM (35 mL) was placed in a salt-ice bath for 10 minutes. After a solution of 3,4,5-trimethoxytoluene (0.55 g, 3 mmol) in DCM (10 mL) was added dropwise, 4-fluorobenzoyl chloride (0.62 g, 3.9 mmol) dissolved in DCM (15 mL) was slowly added dropwise at the same temperature. The resulting yellow solution was warmed to room temperature and stirred for 8 h. The mixture was washed by 0.5 N HCl aqueous (20 mL × 2) and the water layer was extracted with DCM (30 mL) once. The combined DCM layer was washed with sat. Na_2_CO_3_ and water, respectively, dried over anhydrous MgSO_4_, and concentrated to give a crude product which was purified by column chromatography (petroleum: ethyl acetate 3:1, R_f_ = 0.18) to give compound **1a** as a white solid (0.59 g, 65%), mp 114–116 °C; IR (film, cm^−1^) 2939, 2841, 1730, 1670, 1597, 1502, 1332, 1228, 1199, 1151, 1118, 989, 858; ^1^H-NMR (CDCl_3_, 300 MHz) δ (ppm): 2.11 (s, 3H, CH_3_), 3.69 (s, 3H, OCH_3_), 3.85 (s, 3H, OCH_3_), 3.89 (s, 3H, OCH_3_), 6.55 (s, 1H, Ph-H), 7.08–7.15 (m, 2H, Ph-H), 7.81–7.87 (m, 2H, Ph-H). *m/z* (EI) 304 (M^+^). Anal. Calc. for C_17_H_17_FO_4_ (304.31): C, 67.10; H, 5.63; found: C, 67.07; H, 5.66.

*(4-Chlorophenyl)(2,3,4-trimethoxy-6-methylphenyl)methanone* (**1b**). **1b** was prepared from 3,4,5-trimethoxytoluene and 4-chlorobenzoyl chloride using the same procedure as described for **1a** to afford a white solid (69%). mp 99–101 °C (lit. 99.5–100.0 °C) [12]; ^1^H-NMR (CDCl_3_, 400 MHz) δ (ppm): 2.15 (s, 3H, CH_3_), 3.67 (s, 3H, OCH_3_), 3.74 (s, 3H, OCH_3_), 3.83 (s, 3H, OCH_3_), 6.63 (s, 1H, Ph-H), 7.01–7.12 (m, 2H, Ph-H), 7.79–7.86 (m, 2H, Ph-H).

*3,4,5-Trimethoxybenzoic acid*. To a solution of 3,4,5-trimethoxytoluene (1 g, 5.5 mmol) in acetone (20 mL) was added KMnO_4_ (0.76 g, 5.5 mmol) and water (20 mL) at room temperature, followed by 5 drops of concentrated sulphuric acid. More KMnO_4_ (2.28 g, 16.5 mmol) was added in portions during the reaction. The mixture was stirred for 10 h. The precipitate was filtered off and acetone was removed *in vacuo*. The obtained solution was extracted with DCM (30 mL × 2) and the water layer was acidified to pH 2–3 and extracted with DCM (35 mL × 3). The combined DCM layers were dried over anhydrous MgSO_4_, concentrated and crystallized to yield as 3,4,5-trimethoxybenzoic acid as a white solid (0.74 g, 64%).

*Morpholino(3,4,5-trimethoxyphenyl)methanone* (**1c**). To a solution of 3,4,5-trimethoxybenzoic acid (0.21 g, 1 mmol) and morpholine (0.13 g, 1.5 mmol) in DCM (20 mL) was added EDCI (0.23 g, 1.2 mmol) at room temperature. The mixture was stirred for 1 h. The solution was diluted with DCM (40 mL) and washed with water (30 mL × 3). The separated organic layer was dried over anhydrous MgSO_4_, concentrated and crystallized to give **1c** as a white solid (0.17 g, 61%). mp 96–99 °C (lit. 96–98 °C) [14]; IR (film, cm^−1^) 3508, 3492, 2960, 2927, 2852, 1737, 1633, 1583, 1461, 1423, 1326, 1230, 1124, 1002; ^1^H-NMR (CDCl_3_, 400 MHz) δ (ppm): 3.69 (m, 8H, morpholine–H), 3.86 (s, 3H, OCH_3_), 3.88 (s, 6H, 2OCH_3_), 6.63 (s, 2H, Ph–H).

*Benzotriazol-1-yl(2,3,4-trimethoxyphenyl)methanone* (**1d**). To a solution of 3,4,5-trimethoxybenzoic acid (0.21 g, 1 mmol) and 1*H*-benzotriazole (0.14 g, 1.2 mmol) in DCM (20 mL) was added EDCI (0.23 g, 1.2 mmol), followed by the addition of DMAP (0.025 g, 0.2 mmol). The mixture was stirred for 1 h, then diluted with DCM (50 mL) and washed by water (30 mL × 3). The organic layer was dried over anhydrous MgSO_4_ and concentrated. The obtained crude product was purified by column chromatograph (petroleum-ethyl acetate 15:1, R_f_ = 0.15) to give **1d** as a white solid (0.24 g, 77%). mp 119–122 °C (lit. mp 126–128 °C) [15]; IR (film, cm^−1^) 3442, 1737, 1705, 1587, 1450, 1373, 1134, 995, 750; ^1^H-NMR (CDCl_3_, 300 MHz) δ (ppm): 3.96 (s, 6H, 2OCH_3_), 3.99(s, 3H, OCH_3_), 7.55–7.59 (m, 3H, 2Ph–H, BTA-H), 7.73 (t, *J* = 8 Hz, 1H, BTA-H), 8.18 (d, *J* = 8.4Hz, 1H, BTA-H). 8.38 (d, *J* = 8.0 Hz, 1H, BTA-H). *m/z* (EI) 313 (M^+^).

*2-Isopropyl-6-methylpyrimidin-4-yl-3,4,5-trimethoxybenzoate* (**1e**). To a solution of 3,4,5-trimethoxy-benzoic acid (0.21 g, 1 mmol) and 6-hydroxy-2-isopropyl-4-methylpyrimidine (0.15 g, 1 mmol) in DCM (10 mL) was added EDCI (0.28 g, 1.5 mmol). The mixture was stirred for 4 h, then diluted with 90 mL of DCM. The mixture was washed by water and the organic layer was separated, dried over anhydrous MgSO_4_. The solvent was evaporated and the crude product was purified by chromatograph (petroleum-ethyl acetate 10:1, R_f_ = 0.17) on gel silica to give **1e** as a white solid (0.19 g, 55%). mp 97–99 °C; IR (film, cm^−1^): 2970, 2943, 2841, 1739, 1587, 1504, 1415, 1326, 1207, 1128, 974, 750. ^1^H-NMR (CDCl_3_, 400 MHz) δ (ppm): 1.35 (d, *J* = 6.8 Hz, 6H, 2CH_3_), 2.57 (s, 3H, CH_3_), 3.20 (m, 1H, CH), 3.94 (s, 6H, 2OCH_3_), 3.95 (s, 3H, OCH_3_), 6.91 (s, 1H, pyrimidine-H), 7.26 (s, 1H, Ph-H), 7.45 (s, 1H, Ph-H). *m/z* (EI) 346 (M^+^). Anal. Calc. for C_18_ H_22_ N_2_O_5_ (346.38): C, 62.42; H, 6.40; N, 8.09; found: C, 62.43; H, 6.42; N, 8.06.

*4-Morpholinecarbonyl chloride* (**2**). Triphosgene (1.49 g, 5 mmol) was dissolved in DCM (150 mL), then a solution of morpholine (0.87 g, 10 mmol) and triethylamine (1.52 g, 15 mmol) in DCM (30 mL) was slowly added dropwise in a salt-ice bath. After the addition, the reaction was monitored by TLC (iodine vapor detection) until the reaction was completed. Phosgene was blowed off by N_2_, then the mixture was filtered and the filtrate was concentrated to give **2 as** a light-brown oil (1.47 g, 98%, with a content of about 70%), which was used freshly in the next step without further purification.

*Phenylmorpholine-4-carboxylate* (**3a**). To a solution of crude compound **2** (0.96 g, with a content of about 70%, 4.5 mmol), Et_3_N (0.90 g, 9 mmol) and DMAP (0.13 g, 1.1 mmol) in DCM (20 mL) was added a solution of phenol (0.28 g, 3 mmol) in DCM (10 mL) dropwise at room temperature. The mixture was allowed to stir overnight. 100 mL of DCM was added and the mixture was washed successively with sat. Na_2_CO_3_, 0.5 N HCl and brine. The organic layer was dried over anhydrous MgSO_4_, concentrated to give a crude product which was purified by column chromatography (petroleum-ethyl acetate 5:1, R_f_ = 0.30) to give **3a** as a light-yellow solid (0.45 g, 73%). mp 54–56 °C; IR (film, cm^−1^): 2858, 1722, 1419, 1205, 1116, 1064, 856, 742, 690; ^1^H-NMR (CDCl_3_, 300 MHz) δ (ppm): 3.57–3.76 (m, 8H, morpholine–H), 7.10–7.12 (m, 2H, Ph-H), 7.18–7.23 (m, 1H, Ph-H), 7.34–7.39 (m, 2H, Ph-H). *m/z* (EI) 207 (M^+^). Anal. Calc. for C_11_H_13_NO_3_ (207.23): C, 63.76; H, 6.32; N, 6.76; found: C, 63.71; H, 6.36; N, 6.77.

*2-Isopropyl-6-methylpyrimidin-4-yl morpholine-4-carboxylate* (**3b**). To a stirred solution of 6-hydroxy-2-isopropyl-4-methylprimidine (0.15 g, 1 mmol) and 4-morpholinecarbonyl chloride (crude product, 0.28 g, about 1.3 mmol) in DCM (15 mL) was added a solution of Et_3_N (0.20 g, 2 mmol) in DCM (5 mL), followed by addition of DMAP (0.03 g, 0.25 mmol). The mixture was stirred at room temperature for 5 h. DCM (80 mL) was added and the mixture was washed sequentially with cold sat. Na_2_CO_3 _and water. The DCM layer was separated and dried over anhydrous MgSO_4_, concentrated *in vacuo* to give the crude product, which was purified by column chromatography (petroleum-ethyl acetate 3:1, R_f_ = 0.21) to give **3b** as a light-yellow oil (0.19 g, 72%). IR (film, cm^−1^): 2968, 2927, 2864, 1733, 1585, 1421, 1342, 1274, 1230, 1155, 1118, 1060, 848, 746. ^1^H-NMR (CDCl_3_, 300 MHz) δ (ppm): 1.32 (d, *J* = 6.8 Hz, 6H, 2CH_3_), 2.52 (s, 3H, CH_3_), 3.14 (m, 1H, CH), 3.58–3.78 (m, 8H, morpholine-H), 6.86 (s, 1H, pyrimidine-H). *m/z* (EI) 265 (M^+^). Anal. Calc. for C_13 _H_19_N_3_O_3_ (265.31): C, 58.85; H, 7.22; N, 15.84; found: C, 58.78; H, 7.25; N, 15.88.

*1-Phenyl-1H-1,2,4-triazol-3-yl morpholine-4-carboxylate* (**3c**). To a solution of 1-phenyl-1*H*-1,2,4-triazol-3-ol (1.62 g, 10 mmol) in DCM (50 mL) was added K_2_CO_3_ powder (2.07 g, 15 mmol). After the mixture was stirred at r.t. for 0.5 h, a solution of 4-morpholinecarbonyl chloride (crude product, 2.8 g, 13 mmol) in DCM (15 mL) was added, followed by the addition of TBAB (0.64 g, 2 mmol). The mixture was allowed to stir at r.t. overnight. Another 30 mL of DCM was added and the mixture was washed with water (30 mL × 3). The DCM layer was dried over anhydrous MgSO_4_, concentrated in vacuo to give crude product, which was purified by column chromatography (petroleum-ethyl acetate 5:1, R_f_ = 0.31) to give **3c** as a light-yellow solid (2.46 g, 90%). mp 114–115 °C; IR (film, cm^−1^): 3114, 2922, 2862, 1730, 1533, 1415, 1326, 1230, 1109, 1060, 854, 759. ^1^H-NMR (CDC_l3_, 400 MHz) δ (ppm): 3.65–3.78 (m, 8H, morpholine-H); 7.36 (m, 1H, Ph-H); 7.53–7.69 (m, 4H, Ph-H); 9.17 (s, 1H, triazole-H). *m/z* (EI) 274 (M^+^). Anal. Calc. for C_13_H_14_N_4_O_3_ (274.28): C, 56.93; H, 5.14; N, 20.43; found: C, 56.92; H, 5.17; N, 20.40.

*2-Chloro-1-morpholinoethanone* (**4**). To a solution of morpholine (8.7 g, 100 mmol) in THF (250 mL) was added K_2_CO_3_ powder (27.6 g 200 mmol). A solution of chloracetyl chloride (13.6 g, 120 mmol) in THF was added dropwise in an ice-water bath under stirring. After the mixture was stirred for 1 h at the same temperature, the morpholine was converted to a single product nearly quantitatively as monitored by TLC (iodine vapor detection). The reaction mixture was filtered and the filtrate was concentraed *in vacuo*. The residue was dissolved in dichloromethane (200 mL) and washed with sat. aqueous Na_2_CO_3_ and brine, respectively. The organic layer was dried over anhydrous MgSO_4_ and concentrated to give colorless oil which was used in the next steps without further purification.

*General procedure for the preparation of (3,4-dimethoxyphenyl)(4-halogenated-phenyl)methanones* **5a** *and* **5b**. To a suspension of AlCl_3_ (0.81 g, 6 mmol) in DCM (20 mL) was added a solution of 1,2-dimethoxybenzene (0.57 g, 5 mmol) in DCM (10 mL) dropwise in an ice-water bath (0–5 °C), followed by the slow addition of a solution of the appropriate 4-halogenated benzoyl chloride (6 mmol) in DCM dropwise while the temperature was maintained under 10 °C. The mixture was stirred at the same temperature for 2.5 h. DCM (100 mL) was added and the solution was washed successively with 0.5 N aqueous HCl (20 mL) and water. The organic layer was dried over anhydrous MgSO_4_ and filtered. Removal of the solvent gave a white solid which was crystallized from methanol to separately give **5a** and **5b** in yields of 76% and 73%.

*(3,4-Dimethoxyphenyl)(4-halogenated phenyl)methanone oximes* **6a** *and* **6b**. To a refluxing solution of compounds **5a** or **5b** (10 mmol) in anhydrous ethanol was added CH_3_COONa (8.23 g, 100 mmol) and NH_2_OH·HCl (7.03 g, 100 mmol) in portions. The mixture was stirred at reflux for 2.5 h. The reaction was completed and produced only one product as indicated by TLC. After the solid was filtered off, the filtrate was concentrated *in vacuo* to afford a residue, which was dissolved in DCM (250 mL), then washed with water (100 mL × 2). The organic layer was dried over anhydrous MgSO_4_. The solvent was removed *in vacuo* to give **6a** or **6b** as a white solid with the yield of higher than 95%.

*2-((((3,4-Dimethoxyphenyl)(4-fluorophenyl)methylene)amino)oxy)-1-morpholinoethanone* (**7a**). To a solution of **6a** (0.28 g, 1 mmol) and compound **4** (0.17 g, 1 mmol) in THF (30 mL) was added K_2_CO_3_ powder (0.14 g, 1 mmol) and KI (1.66 g, 1 mmol). The mixture was stirred at reflux for 5 h. After the precipitate was filtered off and the solvent in the filtrate was evaporated *in vacuo*, DCM (100 mL) was added to the residue. The resulting solution was washed with water (100 mL × 2), dried over anhydrous MgSO_4_ and concentrated. The obtained crude product was purified by column chromatograph using petroleum-ethyl acetate (5:1) as the eluent to give **7a** as a white solid (0.30 g, 72%). R_f_ = 0.31 (petroleum-ethyl acetate = 5:1, very close to the R_f_ of **7b**); mp 109–112 °C; IR (film, cm^−1^): 3853, 3745, 2958, 2825, 2854, 2360, 1658, 1600, 1442, 1413, 1139, 1022.

*2-((((3,4-Dimethoxyphenyl)(4-chlorophenyl)methylene)amino)oxy)-1-morpholinoethanone* (**7b**) was prepared by the same procedure as described for **7a** as a white solid (0.31 g, 74%). R_f_ = 0.31 (petroleum: ethyl acetate 5:1); mp 112–115 °C; IR (film, cm^−1^): 2690, 2931, 2856, 1666, 1600, 1512, 1234, 1139, 1022, 844; ^1^H-NMR (CDCl_3_, 400 MHz) δ (ppm): 3.42–3.66(m, 8H, morpholine–H), 3.86–3.92 (m, 6H, 2OCH_3_), 4.84 (s, 2H, CH_2_), 6.76–7.03 (m, 3H, Ph-H), 7.10–7.17 (m, 2H, Ph-H), 7.45–7.48 (m, 2H, Ph-H). *m/z* (EI) 418 (M^+^). Anal. Calc. for C_21_H_23_ClN_2_O_5_ (418.87): C, 60.22; H, 5.53; N, 6.69; found: C, 60.25; H, 5.56; N, 6.63.

*(3,4-Dimethoxyphenyl)(4-fluorophenyl)methanone O-morpholine-4-carbonyl oxime* (**7c**). To a solution of **6a** (0.28 g, 1 mmol), Et_3_N (0.15 g, 1.5 mmol) and DMAP (0.03 g, 0.25 mmol) in DCM (15 mL) was added a solution of crude compound **2** (0.32 g, with a content of 70%, 1.5 mmol) in DCM (10 mL) dropwise in ice-water bath. Then the mixture was refluxed for 2 h. DCM (70 mL) was added and the mixture was washed with water. The DCM layer was dried over anhydrous MgSO_4_, concentrated to give a crude product which was purified by column chromatograph (petroleum-ethyl acetate 2:1, R_f_ = 0.26, very close to the R_f_ of **7d**) to give **7c** as a white solid (0.33 g, 85.1%). mp 136–139 °C; IR (film, cm^−1^): 1733, 1600, 1514, 1417, 1232, 1139, 1022.

*(4-Chlorophenyl)(3,4-dimethoxyphenyl)methanone O-morpholine-4-carbonyl oxime* (**7d**). Compound **7d** was prepared by the same procedure as described for **7c** as a white solid (0.36 g, 89.1%). R_f_ = 0.26 (petroleum-ethyl acetate 2:1); mp 130–134 °C; ^1^H-NMR (CDCl_3_, 400 MHz) δ (ppm): 3.21–3.54 (m, 8H, morpholine–H), 3.84 (s, 3H, OCH_3_), 3.95 (s, 3H, OCH_3_), 6.85–6.94 (m, 3H, Ph-H), 7.02–7.07 (m, 2H, Ph-H), 7.58–7.62 (m, 2H, Ph-H). *m/z* (EI) 404 (M^+^). Anal. Calc. for C_20_H_21_ClN_2_O_5_ (404.84): C, 59.33; H, 5.23; N, 6.92; found: C, 59.37; H, 5.24; N, 6.87.

*(3,4-Dimethoxyphenyl)(4-halogenated)methanols* **8a** *and* **8b**. To a solution of compound **5** (10 mmol) in DCM (40 mL) and methanol (10 mL) was added NaBH_4_ powder (0.57 g, 15 mmol) in portions at room temperature. The mixture was refluxed for 1 h. The mixture was poured into a beaker and quenched by addition of cold 0.2 N HCl. After the precipitate was filtered off, the filtrate was extracted with DCM (100 mL × 2). The combined organic layer was washed with brine, dried over anhydrous MgSO_4_, filtered and concentrated *in vacuo* to give **8a** or **8b** as a white powder in nearly quantitative yield that was used directly for the next steps.

*2-((3,4-Dimethoxyphenyl)(4-fluorophenyl)methoxy)-1-morpholinoethanone* (**9a**). To a solution of **8a** (0.52 g, 2 mmol) in THF (25 mL) was added NaH (76%, 0.13 g, 4 mmol), followed by the addition of a solution of **4 **(0.33 g, 2 mmol) in THF (10 mL) dropwise at room temperature. The mixture was stirred for 2 h. The mixture was filtered and filtrate was concentrated. The residue was dissolved in DCM (150 mL) and washed with water. The organic layer was dried over anhydrous MgSO_4_, filtered and concentrated *in vacuo* to give a crude product, which was purified by column chromatography on silica gel (petroleum-ethyl acetate 3:1, R_f_ = 0.13) to give **9a** as a colorless oil (0.71 g, 91.6%). IR (film, cm^−1^): 3531, 2931, 2856, 1651, 1508, 1463, 1261, 1234, 1112, 1026, 846; ^1^H-NMR (CDCl_3_, 400 MHz) δ (ppm): 3.46–3.67 (m, 8H, morpholine–H), 3.83(s, 3H, OCH_3_), 3.87 (s, 3H, OCH_3_), 4.09–4.18 (s, 2H, CH_2_), 5.45 (s, 1H, CH), 6.81–6.86 (m, 3H, Ph-H), 6.99–7.03 (m, 2H, Ph-H), 7.26–7.33 (m, 2H, Ph-H). *m/z* (EI) 389 (M^+^). Anal. Calc. for C_21_H_24_FNO_5_ (389.42): C, 64.77; H, 6.21; N, 3.60; found: C, 64.81; H, 6.20; N, 3.57.

*2-((4-Chlorophenyl)(3,4-dimethoxyphenyl)methoxy)-1-morpholinoethanone* (**9b**) The title compound was prepared using the same procedure as described for **9a** to give a light-yellow oil (83.9%). R_f_ = 0.13 (petroleum-ethyl acetate 3:1); IR (film, cm^−1^): 3491, 2956, 2925, 2854, 1651, 1510, 1461, 1263, 1234, 1112, 1026, 846; ^1^H-NMR (CDCl_3_, 400 MHz) δ (ppm): 3.46–3.67 (m, 8H, morpholine–H), 3.83 (s, 3H, OCH_3_), 3.87 (s, 3H, OCH_3_), 4.09–4.18 (m, 2H, CH_2_), 5.45 (s, 1H, CH), 6.81–6.86 (m, 3H, Ph-H), 6.99–7.01 (m, 2H, Ph-H), 7.26–7.33 (m, 2H, Ph-H). *m/z* (EI) 405 (M^+^). Anal. Calc. for C_21_H_24_ClNO_5_ (405.87): C, 62.14; H, 5.96; N, 3.45; found: C, 62.19; H, 5.92; N, 3.43.

*(4-Halogenated phenyl)(3,4-dimethoxyphenyl)methanamines* **10a** *and* **10b**. To a solution of compound **6a** or **6b** (2 mmol) in ethanol (10 mL) was added a 3% aqueous solution of ammonia, NH_4_OAC (0.26 g, 3.6 mmol) and water (10 mL). The mixture was refluxed and Zn powder (0.65 g, 10 mmol) was added in portions during the reaction, after being refluxed for 6 h, the solid was filtered off. The solvent in the filtrate was evaporated, and DCM (100 mL) and water (100 mL) were added. The DCM layer was separated, washed with water, dried over MgSO_4_ and concentrated to give **10a** or **10b** as a colorless and viscous oil (0.47 g, 91% for **10a**, 0.51g, 92% for **10b**), which was used for the next step without further purification.

*2-(((4-Fluorophenyl)(3,4-dimethoxyphenyl)methyl)amino)-1-morpholinoethanone* (**11a**). To a mixture of **10a** (0.26 g, 1 mmol), K_2_CO_3_ (0.24 g, 2 mmol) and KI (0.17 g, 1 mmol) in CH_3_CN (30 mL) was added dropwise a solution of compound **4** (2.3 g, 1.5 mmol) in CH_3_CN (10 mL). The mixture was stirred at room temperature for about 15 h. After the solid was filtered off, the solvent in the filtrate was removed *in vacuo*. The residue was dissolved in DCM (100 mL) and water (100 mL). The organic layer was washed with brine (50 mL × 2), dried over anhydrous MgSO_4_, filtered and concentrated to give a crude product which was purified by column chromatography (petroleum-ethyl acetate 1:3, R_f_ = 0.27) to afford **11a** as a white powdery solid (0.29 g, 72%). mp 167–170 °C; IR (film, cm^−1^): 3550, 3492, 2958, 2923, 2854, 1651, 1456, 1232, 1114, 1029, 848, 732; ^1^H-NMR (CDCl_3_, 300 MHz) δ (ppm): 3.23–3.34 (m, 4H, morpholine-H), 3.54–3.65 (m, 7H, morpholine-H, -CH_2_, -CH), 3.84 (s, 3H, OCH_3_), 3.85 (s, 3H, OCH_3_), 5.35 (m, 1H, -NH), 6.78 (d, *J* = 11.2 Hz, 1H, Ph-H), 6.91 (d, *J* = 11.2 Hz, 1H, Ph-H), 6.95–7.03 (m, 3H, Ph-H), 7.40–7.46 (m, 2H, Ph-H). *m/z* (EI) 388 (M^+^). Anal. Calc. for: C_21_H_25_FN_2_O_4_ (388.43): C, 64.93; H, 6.49; N, 7.21; found: C, 64.95; H, 6.45; N, 7.23.

*2-(((4-Fluorophenyl)(3,4-dimethoxyphenyl)methyl)amino)-1-morpholinoethanone* (**11b**). The title compound was prepared as a white solid (0.29 g, 71%) by the same procedure as described for **11a**. R_f_ = 0.27 (petroleum-ethyl acetate 1:3); mp 179–181 °C; IR (film, cm^−1^): 2956, 2923, 2854, 1643, 1506, 1454, 1267, 1232, 1114, 1029, 848.

*N-((4-Chlorophenyl)(3,4-dimethoxyphenyl)methyl)morpholine-4-carboxamide* (**11c**). To a solution of compound **10b** (0.28 g, 1 mmol) in DCM (15 mL) was added Et_3_N (0.15 g, 1.5 mmol) and DMAP (0.025 g, 0.2 mmol), followed by the addition of a solution of compound **2** in DCM (5 mL). The mixture was stirred at room temperature overnight, then DCM (60 mL) was added. The organic layer was washed with water (50 mL × 2) and dried over anhydrous MgSO_4_. The DCM was evaporated *in vacuo* to give a crude product which was purified by column chromatography (petroleum- ethyl acetate 1:1, R_f_ = 0.34) to give compound **11c** as a white solid (0.28 g, 71.8%). mp 90–93 °C; IR (film, cm^−1^): 2956, 2923, 2852, 1627, 1510, 1461, 1253, 1116, 1026, 831, 746, 657; ^1^H-NMR (CDCl_3_, 400 MHz) δ (ppm): 3.36–3.40 (m, 4H, morpholine–H), 3.67–3.71 (m, 4H, morpholine-H), 3.81 (s, 3H, OCH_3_), 3.85 (s, 3H, OCH_3_), 4.88 (d, *J* = 6.6Hz, 1H, CH), 6.06 (d, *J* = 6.6 Hz, 1H, NH), 6.73–6.83 (m, 3H, Ph-H), 6.98–7.03 (m, 2H, Ph-H), 7.20–7.26 (m, 2H, Ph-H). *m/z* (EI) 390 (M^+^). Anal. Calc. for: C_20_H_23_ClN_2_O_4_ (390.86): C, 61.46; H, 5.93; N, 7.17; found: C, 61.49; H, 5.95; N, 7.12.

### 3.2. Biological Tests

#### 3.2.1. Antifungal Methods

The synthetic compounds were dissolved in DMSO and sterilized water (containing 1% Tween) and diluted with PDA in a Petri dish. The antifungal activity was tested *in vitro* by the poisoned food technique [22,23] at a final concentration of 50 μg/mL. The widely used and commercial available fungicides dimethomorph and flumorph were used as positive controls.

#### 3.2.2. Antibacterial Methods

About 10 mL of sterilized 2216E medium was poured into the sterilized Petri dish. After solidification, 1 mL of inoculum of each test vibrio was added and sprayed uniformly. Then a 4 mm well was made in the centre of each plate using a sterilized cork-borer. Each well received 20 μL of solution of the tested compounds dissolved in DMSO. The diameters were measured after the plates were incubated at 28 °C for about 24 h. Two antibiotics, streptomycin and gentamicin sulfate were used as the positive controls. The blank control (DMSO) was of no inhibition to the vibrios.

## 4. Conclusions

A series of benzophenone compounds was designed and synthesized. The biological activity evaluation indicated that the synthesized compounds were quite potent on most of the tested fungi, but much less effective on the five tested vibrios. At least one of the novel benzophenone derivatives gave higher activity than flumorph and dimethomorph towards the tested fungi. Compounds **1a** and **1b** exhibited relatively good activity against five fungi, making them interesting leads for the design and optmization of new fungicides. Six compounds (**1c**, **1e**, **3b**, **3c**, **7c** and **11a**) had effects on all of the tested fungi from different genera and showed better wide-spectrum activity. A Cl atom, three methoxy groups on the benzene ring and a pyrimidine group might be favorable for increasing the antifungal activity. The screened compound **7b** might be a better candidate than carbendazim to inhibit *Fusarium oxysporum f. sp. vasinfectum.* The structure-activity relationships were discussed preliminarily and should be somewhat helpful for further studies to design more potent derivatives and discovery better fungicides with different modes of action.

## Supplementary Information

Supplementary materials can be accessed on: http://www.mdpi.com/1420-3049/16/11/9739/s1.
