# Supplementary figures and images for: Diversity Oriented Design of Various Benzophenone Derivatives and Their in Vitro Antifungal and Antibacterial Activities
Source: Molecules. 2011 Nov 23;16(11):9739–54. doi: 10.3390/molecules16119739 (PMC6264486; doi:10.3390/molecules16119739)

**Supplementary Materials**

**1. IR：**

**1a**

**1c**

**1d**

**1e**

**3a**

**3b**

**3c**

**7a**

**7b**

**7c**

**9a**

**9b**

**11a**

**11b**

**11c**

**2. H-NMR：**

**1a**


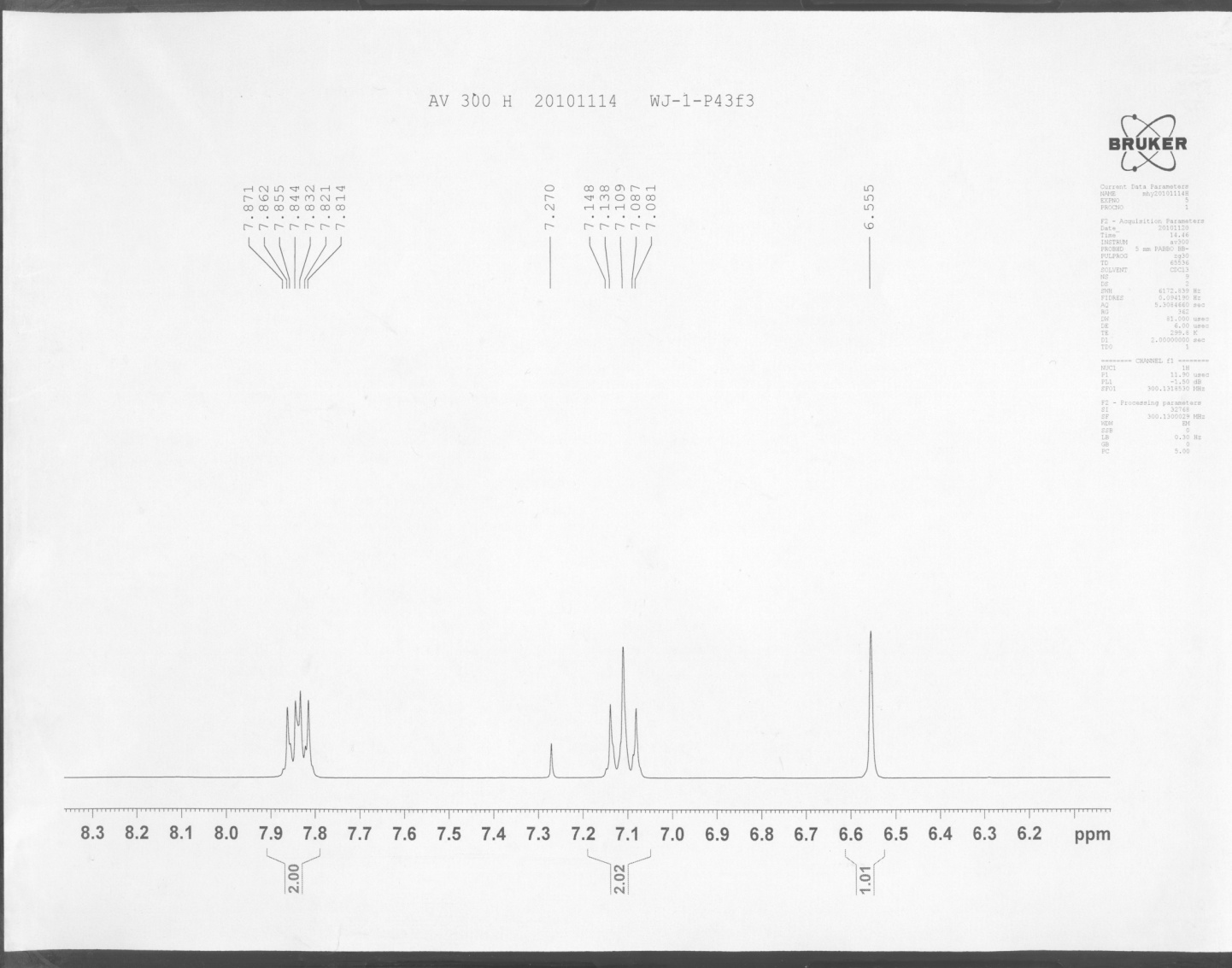


**1c**


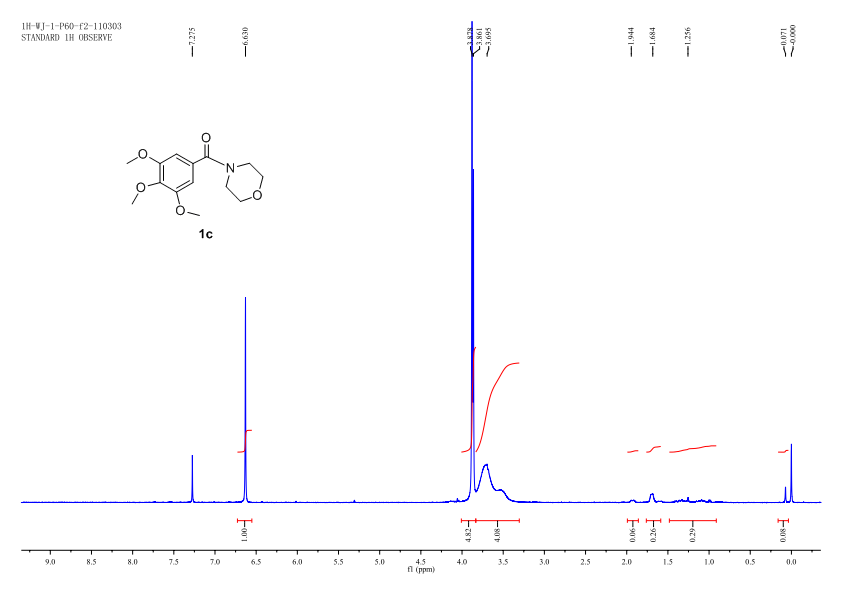


**1d**


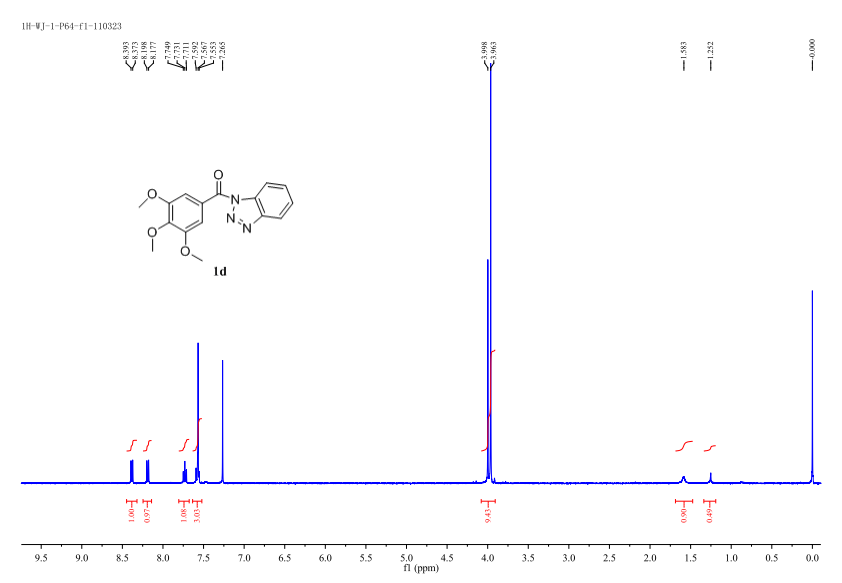


**1e**


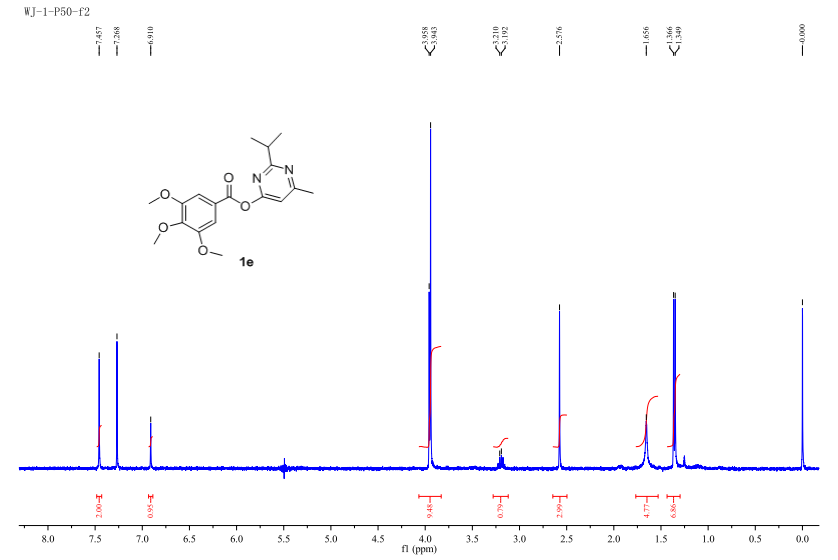


**3a**


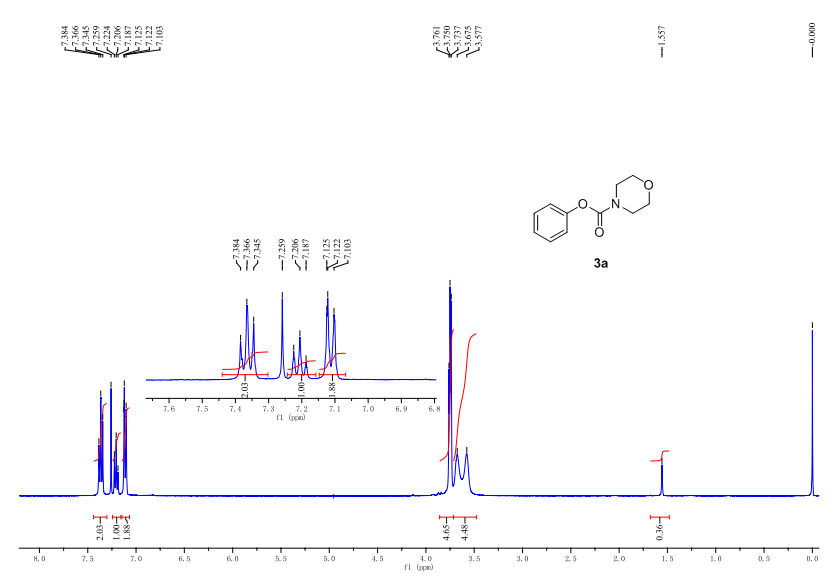


**3b**


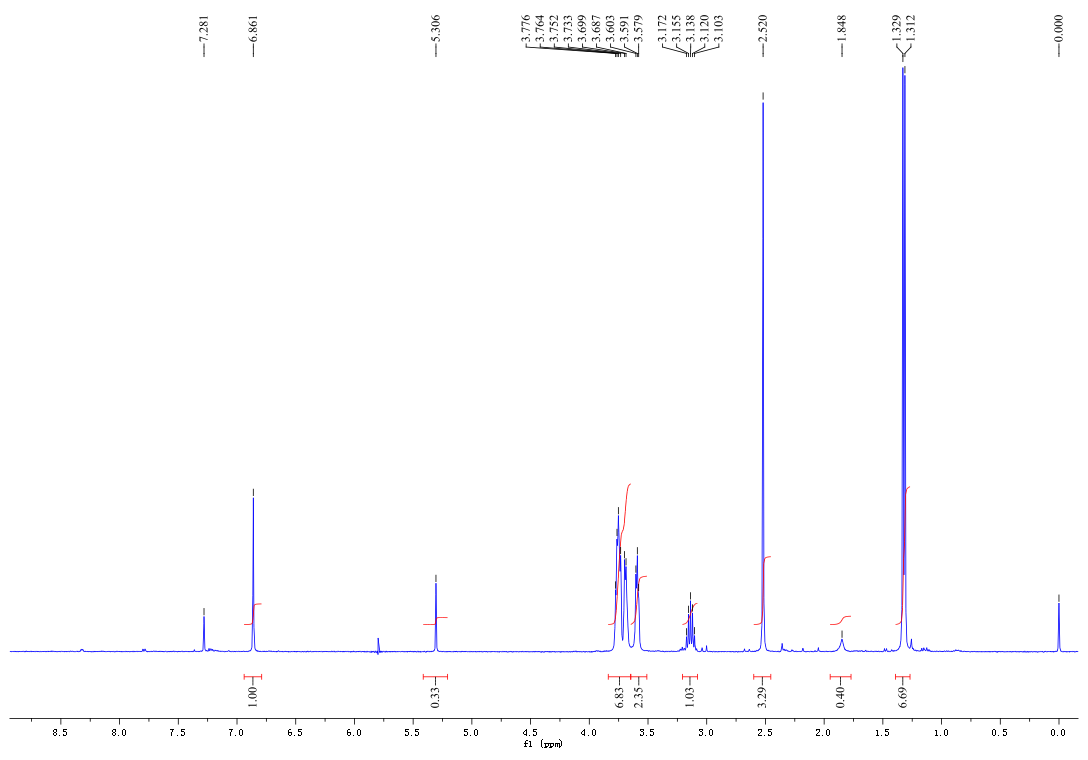


**7b**


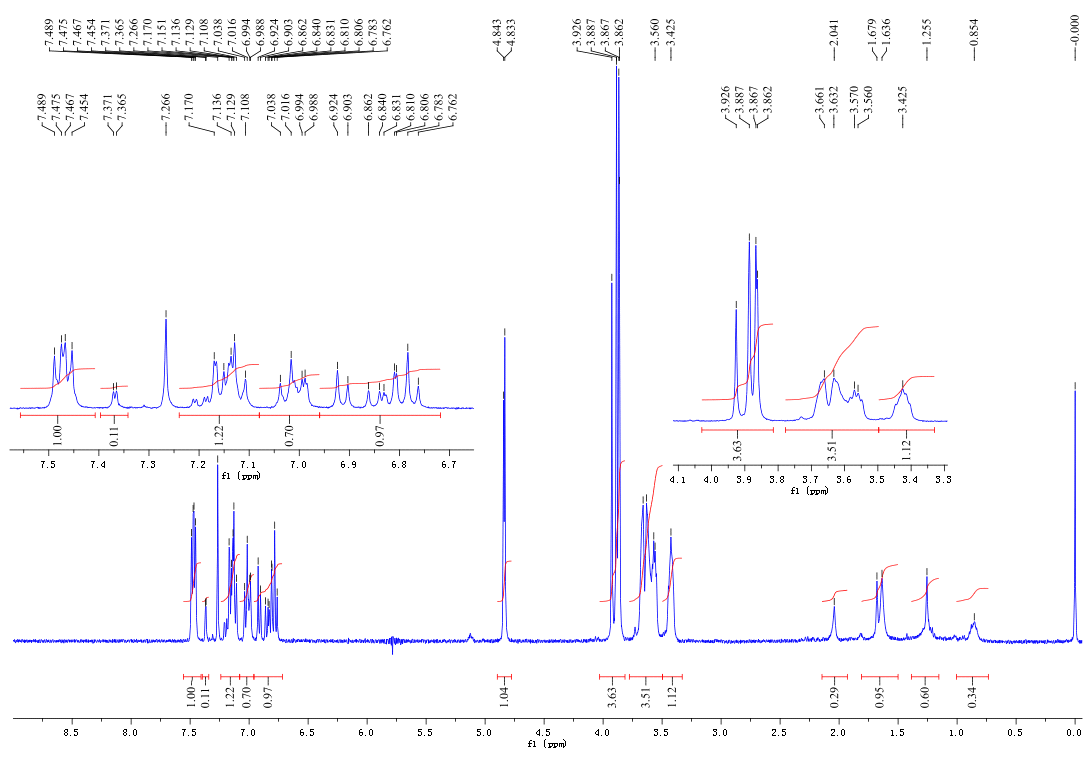


**7d**

**9a**


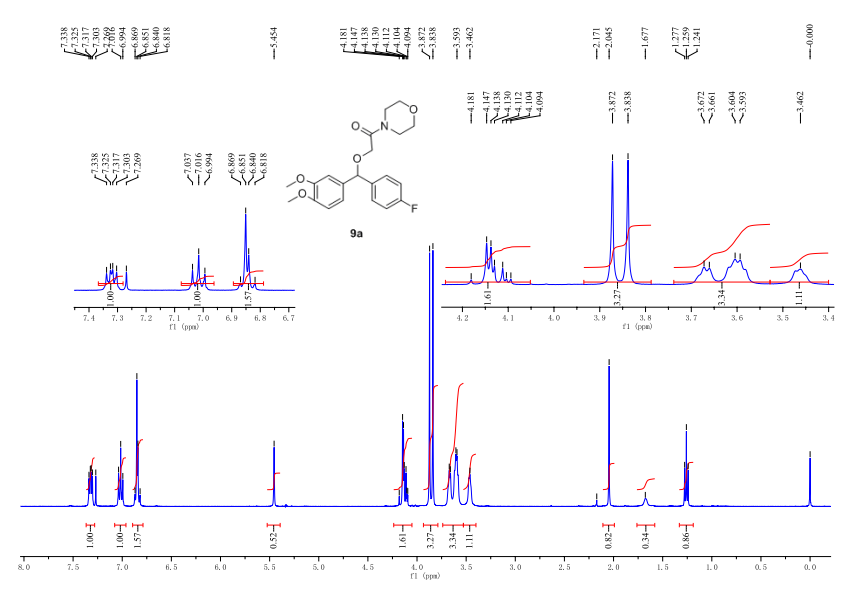


**9b**

**
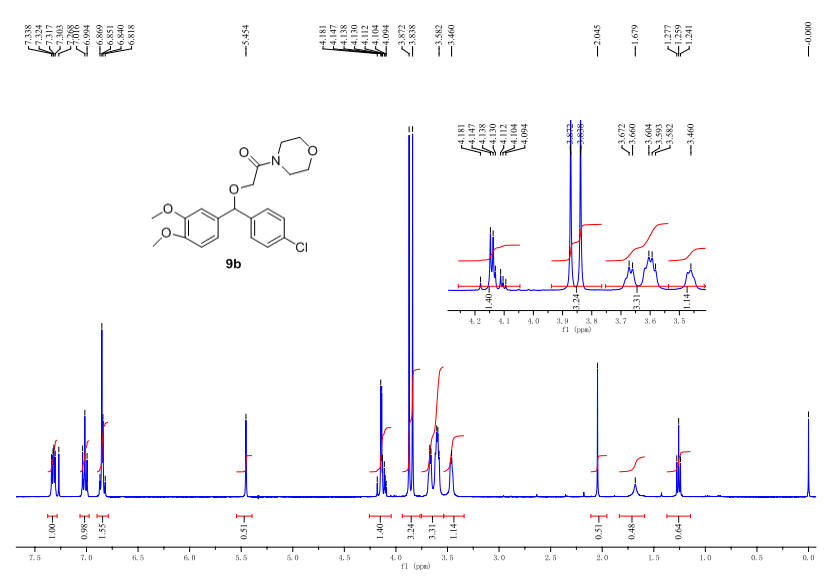
**

**11a**


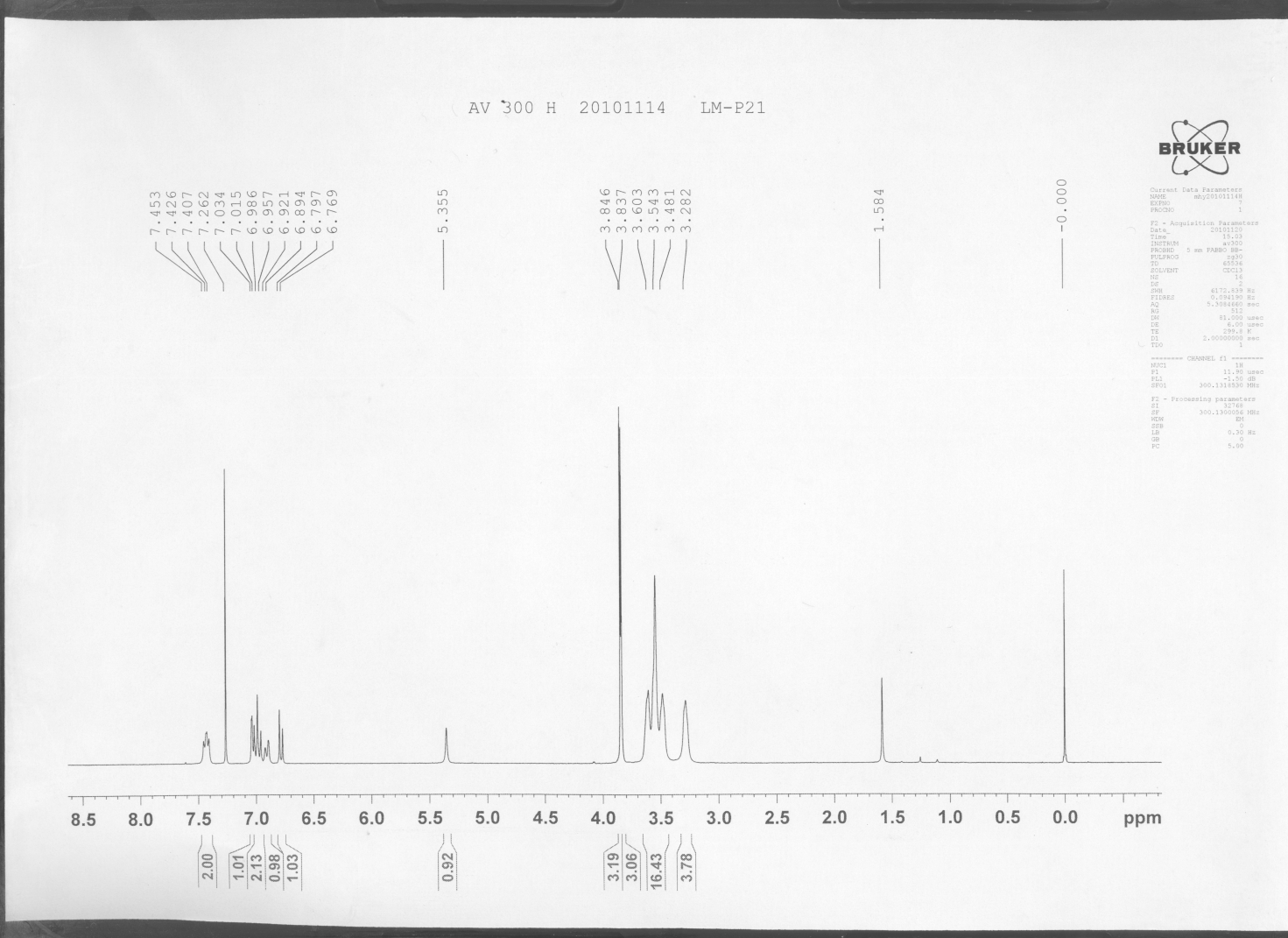


**11c**


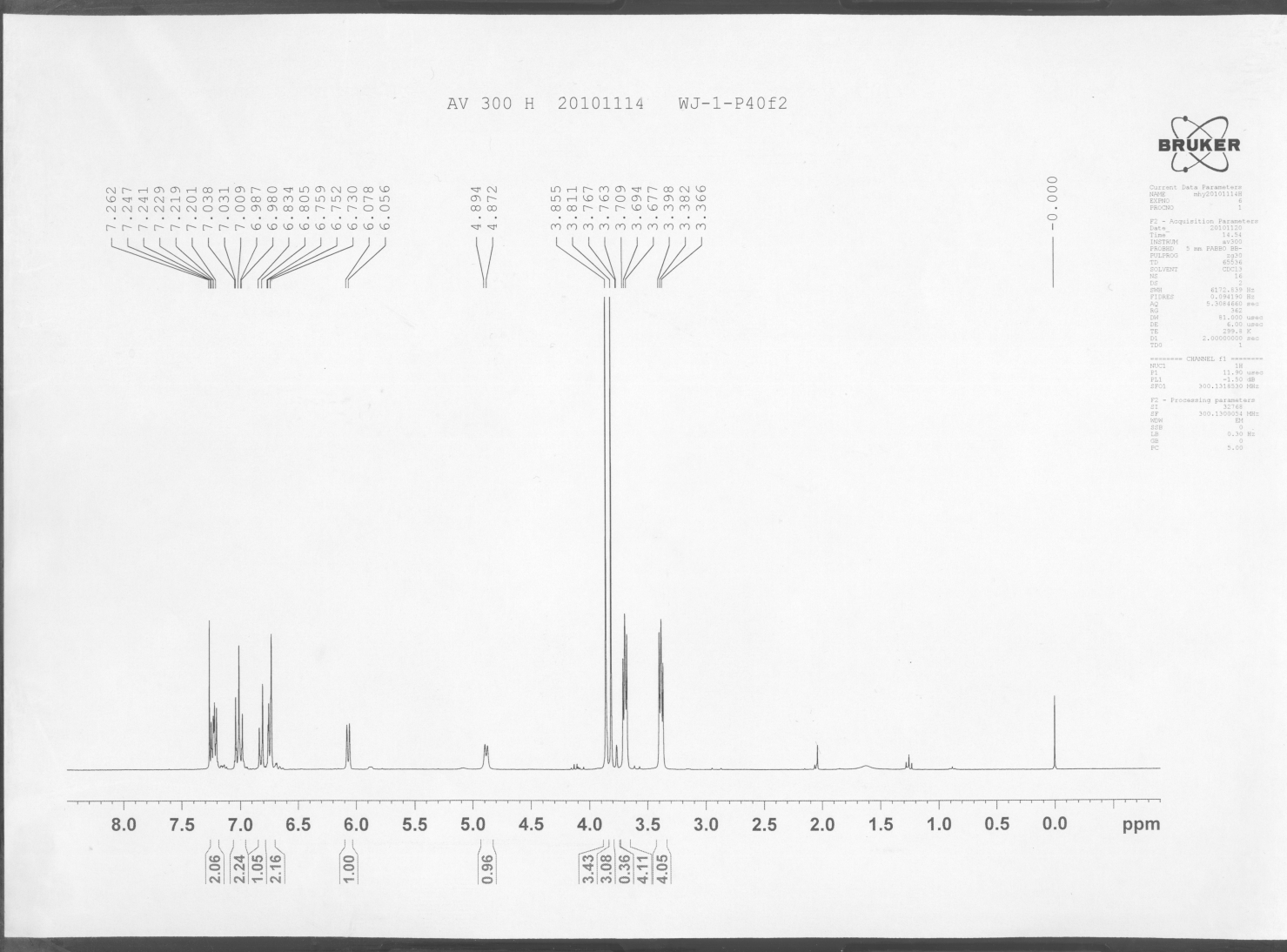

Supplement: Supplementary file 1 [file molecules-16-09739-s001.docx]
